# Effect of feeding hydroponic barley seedlings to lactating ewes on blood biochemical indexes and growth performance of lambs

**DOI:** 10.3389/fvets.2023.1280544

**Published:** 2024-01-05

**Authors:** Yan Ma, Tongjun Guo, Zhijun Zhang, Guzalnur Amat, Yaxing Jing, Yong Tuo, Liangzhong Hou

**Affiliations:** ^1^Feed Research Institute, Xinjiang Academy of Animal Sciences, Urumqi, China; ^2^Key Laboratory of Xinjiang Feed Biotechnology, Urumqi, China

**Keywords:** hydroponic barley seedlings, lactating Hu ewes, lambs, milk quality, antioxidants

## Abstract

The aim of this study was to investigate the effects of replacing different ratios of basal diets with hydroponically barley seedlings (HBS) on the serum biochemical indexes and growth performance of lambs. It provides a theoretical basis for the use of HBS in ruminant health and scientific feeding management. In total, 30 ewes were randomly categorized into six groups (two control groups, 4 treatment groups, and 8 replicates in each group). All experiments were conducted under the same feeding and management conditions, on this basis the control group was CK1 and CK2 groups, which CK1 group added 15% corn silage, the treatment groups replacing 5% (group A), 10% (group B), 15% (group C) and 20% (group D) of the basal diet (dry matter basis) with HBS, and the experimental period lasted for 36 days, and the lambs were lactating with their mothers throughout the experimental period. Key results. The contents of total protein (TP), albumin (ALB), milk fat percentage and total solids (TS) in group C were significantly higher than CK1 and CK2 groups (*p* < 0.05) in milk samples; malondialdehyde (MDA) content in groups A and C was significantly lower than groups CK1 and CK2 (*p* < 0.05), alanine aminotransferase (ALT) and azelaic transaminase (AST) contents in groups A and B were significantly higher than CK1 group (*p* < 0.05), TC content in groups A and D was significantly higher than CK1 and CK2 groups (*p* < 0.05), high-density lipoprotein (HDL-c) content in group D was significantly higher than CK1 and CK2 groups (*p* < 0.01) in blood samples; Body height in C group was significantly higher than CK2 group (*p* < 0.05), ear width in group B was significantly higher than CK1 group (*p* < 0.05). In conclusion, under the conditions of this experiment, HBS instead of 5–15% of the basal diet could improve the milk quality of lactating Hu ewes and alleviate the oxidative stress of the body.

## 1 Introduction

With the continuous expansion of animal husbandry breeding scale, feed shortage has become one of the restrictive factors for the healthy development of the industry. Therefore, it is urgent to accelerate the development and use of unconventional feeds to alleviate the shortage of feed resources. The dry matter content (DM) of hydroponic barley grass is 10.17%, the crude protein (CP) content is 14.98%, the crude fat (EE) content is 3.1%, the calcium (Ca) content is 0.24%, and the phosphorus (P) content is 0.40%. The crude protein, crude fat, and phosphorus content are all higher than that of barley hay (1). Moreover, during the growth period of hydroponic barley grass, the dry matter and starch content decreases with the culture time, and starch is metabolized into soluble polysaccharides to support metabolic and energy needs. The total content of mineral elements, vitamins, and amino acids increases with the increasing culture time (2). As the germination process of barley grains progresses, the content of water-soluble proteins increases, and the total amino acid content also increases significantly (3, 4). The protein and starch involved in the decomposition process involve multiple digestive hydrolytic enzymes, which can enhance the body’s digestive function and improve appetite problems (5). By measuring the nutritional value of barley seedlings under different hydroponic time, it was found that its dry matter and starch content decreased with the increase of hydroponic time, and the content of mineral elements such as calcium (Ca), phosphorus (P), potassium (K), magnesium (Mg), crude protein content and crude fat content increased. Magnesium ions mainly act as enzyme activation, regulate calcium and potassium ion channels and promote bone growth (6). Calcium ions are mainly involved in the construction of bones, and potassium ions are mainly involved in maintaining the osmotic pressure (7). Acid–base balance and glycometabolism of the intracellular fluid in animal cells (8).

Barley seedling cultivated by hydroponic technology can be used as a green feed to alleviate the shortage of animal feed resources, grassland degradation and water shortage in pastoral areas. Research has shown that hydroponic barley sprouts have a high vitamin content, which can improve the lactation ability of herbivorous livestock. Harvested hydroponic barley sprouts can provide vitamin E to herbivorous livestock, which has a positive effect on their cell antioxidant capacity and reproductive performance (9). Devendar et al. (10) found that replacing 50% of mixed concentrate with hydroponic barley sprouts in lamb diets can improve their growth performance. Replacing 30% of barley grains can also improve certain rumen characteristics, and increase the digestibility and feed conversion rate of most nutrients. Fazaeli et al. (11) found that replacing 20, 40, and 60% of corn silage with hydroponic barley sprouts in dairy cow diets had no significant effect on their average daily milk production or lactation performance. In conclusion, hydroponic barley sprouts can meet the nutritional needs of herbivorous animals and have the potential to be developed into high-quality forages (12, 13), but the available research on using hydroponic barley sprouts as livestock feed, especially during lactation in sheep, is limited. Therefore, this study selected lactating Hu lambs as the experimental subjects to investigate the effects of different proportions of hydroponic barley sprouts replacing basic feed on serum biochemical indexes and growth performance, providing a reference for further research on the feeding value of hydroponic barley sprouts in sheep production.

## 2 Materials and methods

### 2.1 Test animals and materials

Hu ewes were used as test animals in this experiment, which were provided by the Hu ewes breeding base of Xinjiang Mai teng Herding Science and Technology Development Co. HBS were cultivated by Xinjiang Green Chuangfeng Agricultural Development Co. and the growing period was 7 days.

### 2.2 Experimental design and diet composition

Forty-eight healthy lactating Hu ewes with similar age (2–3 years), litter size (2–3), body weight (45 ± 5 kg), and two lambing number, were selected for the experiment, and were divided into six groups by a one-way completely randomized experimental design, with two control groups of eight replicates each and four treatment groups of eight replicates each. The experimental diets were based on the NRC (2007) 45 kg with double lamb ewes lactation nutritional requirements, All experiments were conducted under the same feeding and management conditions, the control groups was CK1 and CK2 groups, which CK1 group added 15% corn silage, and the treatment group diets were used in an equal replacement method by replacing 5% (group A), 10% (group B), 15% (group C), and 20% (group D) of the basal diet (dry matter basis) with hydroponically grown barley seedlings, respectively (Table 1).

**Table 1:** Experimental diet composition and nutritional level (in dry matter basis).

Items	Groups					
	CK^1^	CK^2^	A	B	C	D
Composition of raw materials/%						
Corn silage	15.00	–	–	–	–	–
Hydroponic barley seedlings	0.00	0.00	5.00	10.00	15.00	20.00
Halm	21.60	26.20	24.89	23.58	22.27	20.80
Alfalfa 17% CP	10.10	15.00	14.25	13.50	12.75	12.00
Mixed concentrate	52.80	58.30	55.36	52.42	49.48	46.70
Premix1	0.50	0.50	0.50	0.50	0.50	0.50
Total	100.00	100.00	100.00	100.00	100.00	100.00
Nutritional level						
ME (MJ/kg)	10.09	10.09	10.13	10.17	10.21	10.25
CP (g/kg)	128.45	128.40	122.93	117.45	111.98	106.50
NDF (%)	39.93	33.26	33.02	32.77	32.53	32.29
Ca (g/kg)	4.68	4.56	4.36	4.15	3.95	3.75
P (g/kg)	3.14	2.90	2.79	2.69	2.58	2.48

^1^The premix provides per kg of concentrate supplement: vitamin A 4200 IU, vitamin B1 0.4 mg, vitamin B22 mg, vitamin B6 1.2 mg, vitamin C 20 mg, vitamin D3 880 IU, vitamin E 500 IU, pantothenic acid 10 mg, niacinamide 100 mg, copper 25 mg, iron 107 mg, manganese 81 mg, zinc 74 mg, and Iodine 6 mg, Selenium 14 mg, Cobalt 3 mg, Choline Chloride 120 mg. ^2^Nutritional levels are measured.

### 2.3 Feeding management

Before the beginning of the test, the sheep pens were sterilized and disinfected, and all the test lake sheep (big sheep) were orally dewormed with Ivermectin before the test, and ear numbers were marked. The whole animal feeding test was carried out in the same environment by house feeding, and the diets of each group were accurately weighed and evenly mixed according to the proportion of the formula, and then fed regularly at 9:00 and 17:00 every day, and the test period was 36 days, of which the pre-feeding period was 6 days, and the official period was 30 days, and the lambs nursed with the ewes throughout the whole period of the test, and they could freely feed on ewes’ materials and drink water freely.

### 2.4 Sample collection and indicator measurement

#### 2.4.1 Sample collection and processing

(1) At the end of the 31 days feeding period of the formal test, collect the blood of lambs through the jugular vein into sodium heparin anticoagulant tubes, centrifuge at 3,000 rpm/min for 15 min at 4°C, take the supernatant, and separate it into 1.5 mL centrifuge tubes (3 tubes), and store it at −20°C, which is mainly used for the analysis of the immune indexes in the blood.
(2) On the 28th to 30th day of the official test, milk samples were collected from test ewes by hand milking, and the ewes milk were collected three times in the morning at 9:00 a.m., in the middle at 14:00 a.m. and in the evening at 20:30 p.m. in the ratio of 4:3:3, and then packed in 50 mL ewes milk bottles, immediately send to the testing company to determine the relevant indexes.
(3) All test lambs were weighed and recorded on a hanging scale before morning feeding on the 1st and 31st days of the test to calculate the average total weight gain and average daily weight gain of the lambs; the body size of the lambs was measured on the 0 and 30th days of the positive test period before morning feeding. During the measurement, the lambs were pulled to the flat ground in a natural standing position, and the body height and body length of the lambs were measured with a measuring stick, and the chest circumference, chest depth, chest width, tube circumference, ear length, ear width, and cross section height were measured with a tape measure and recorded.

#### 2.4.2 Measurement of sample indexes

Ewes milk composition indexes: total protein (TP), albumin (ALB), β-globulin (β-AFP), lactoferrin (LTF), lysozyme (LZM), lactose content, milk fat rate, total solids (TS).

Antioxidant indexes of ewes milk: antioxidant indexes: malondialdehyde (MDA), total superoxide dismutase (T-SOD), glutathione peroxidase (GSH-Px), total antioxidant power (T-AOC), which were determined by Nanjing Aoqing Biotechnology Co.

Plasma biochemical indexes: glucose (GLU), creatine kinase (CK), lactate dehydrogenase (LDH), alanine aminotransferase (ALT), azelaic transaminase (AST), cholesterol (TC), triglyceride (TG), low-density lipoprotein (LDL), high-density lipoprotein (HDL), total protein (TP), urea nitrogen (BUN).

Immune indexes: interleukin-1β (IL-1β), interleukin-2β (IL-2β), interferon gamma (IFN-γ), CD4-T lymphocytes (CD4); antioxidant indicators: malondialdehyde (MDA), total superoxide dismutase (T-SOD), and glutathione peroxidase (GSH-Px), as determined by Nanjing Jianjian Bioengineering Institute.

### 2.5 Data processing

The data of blood samples, milk samples, body weight and body measurements were preliminarily organized by Excel 2010, and the ANOVA procedure of SPSS 24.0 statistical software was used for one-way ANOVA, while significant differences were used for multiple comparisons by Duncan’s method, and the results of the test were expressed as the mean (MEAN) and standard error of the mean (SEM), and *p* < 0.05 as significant level of difference, and *p* < 0.01 as highly significant level of difference.

## 3 Results and analysis

### 3.1 Effect of HBS substituting different proportion of basal diet on protein content of ewes milk

As shown in Table 2, the content of TP in group C was significantly or highly significantly higher than groups CK1 and CK2 increased by 18.97 and 15.42%, respectively (*p* < 0.01 or *p* < 0.05), and group D was highly significantly higher than group CK1 increased by 18.97% (*p* < 0.01); ALB in groups C and D was significantly higher than groups CK1 and CK2 (*p* < 0.05); There was no significant differences in β-AFP, LTF, and LZM contents among groups (*p* > 0.05).

**Table 2:** Effect of HBS substituting different proportion of basal diet on protein content of ewes milk.

Items	Groups							
	CK1	CK2	A	B	C	D	SEM	*P*
TP g/L	40.27^Cc^	41.51^BCbc^	44.87^ACBcba^	45.70^ABCab^	49.84^Aa^	47.91^ABa^	0.904	0.004
ALB g/L	21.00^b^	22.44^b^	33.41^ab^	28.41^ab^	35.60^a^	39.74^a^	2.009	0.020
β-AFP g/L	4.05	5.31	5.35	5.70	5.09	5.31	2.016	0.925
LTF g/L	171.87	163.31	178.14	186.66	218.17	177.32	34.725	0.897
Lysyme μg/mL	132.90	210.88	185.00	158.33	232.06	203.46	25.314	0.911

Different lowercase letters show significant differences after peer data (*P* < 0.05), and different uppercase letters show significant differences after peer data (*P* < 0.01). The same as Tables 3–10.

### 3.2 Effect of HBS substituting different proportion of basal diet on antioxidant capacity of ewes milk

As shown in Table 3, the content of GSH-PX in group B was significantly or highly significantly higher than group CK1 increased by 27.85% (*p <* 0.01 or *p* < 0.05); There was no significant differences in T-AOC, SOD and MAD contents among groups (*p >* 0.05).

**Table 3:** Effect of HBS substituting different proportion of basal diet on antioxidant capacity of ewes milk.

Items	Groups							
	CK1	CK2	A	B	C	D	SEM	*P*
T-AOC U/mL	7.23	6.26	7.59	5.48	8.42	7.79	0.602	0.791
SOD U/mL	482.47	522.41	584.58	546.26	640.08	613.04	35.011	0.835
GSH-px U/mL	170.32^Bd^	184.85^ABcd^	213.74^Aab^	217.75^Aa^	208.08^Aabc^	190.84^ABbcd^	4.545	0.003
MAD nmol/mL	1.96	1.94	2.15	2.98	2.03	2.09	0.176	0.560

### 3.3 Effect of HBS substituting different proportion of basal diet on milk composition content

As shown in Table 4, the content of Lactose in group A was extremely significantly higher than groups CK1 and CK2 increased by 34.55 and 27.54%, respectively (*p* < 0.01); Milk fat percent in Groups A, C, and D were significantly higher than group CK2 increased by 28.25, 32.69, and 25.71%, respectively (*p* < 0.05); TS in groups A and C were significantly higher than group CK1, increased by 12.65 and 16.36%, respectively (*p* < 0.05).

**Table 4:** Effect of HBS substituting different proportion of basal diet on milk composition content.

Items	Groups							
	CK1	CK2	A	B	C	D	SEM	*P*
Lactose g/100 mL	3.82^Cd^	4.03^BCcd^	5.14^Aa^	4.62^ABCabc^	4.86^ABab^	4.35^ABCbcd^	0.120	0.002
Milk fat percent %	3.43^ab^	3.15^b^	4.04^a^	3.59^ab^	4.18^a^	3.96^a^	0.114	0.045
Total solids %	16.20^Cc^	16.93^BCc^	18.25^ABab^	17.10B^Cbc^	18.85^ABa^	16.88^Cc^	0.238	0.002

### 3.4 Effects of HBS substituting different proportion of basal diet on plasma antioxidant capacity of lambs

As shown in Table 5, the content of MAD in groups A and C was significantly lower than groups CK1 (*p* < 0.05); and there was no significant effect on the SOD and GSH-px contents among the groups (*p* > 0.05).

**Table 5:** Effects of hydroponic barley seedling substituting different proportion of basal diet on plasma antioxidant capacity of lambs.

Items	Groups							
	CK1	CK2	A	B	C	D	SEM	*P*
SOD U/mL	171.98	167.73	165.17	170.21	154.45	165.13	2.898	0.608
GSH-px U/mL	27.49	23.20	29.22	28.88	20.87	22.64	1.069	0.079
MAD nmol/mL	7.32^Aa^	6.21^ABab^	4.50^Bc^	5.56^ABbc^	4.81^Bc^	5.79^ABbc^	0.228	0.009

### 3.5 Effects of HBS substituting different proportion of basal diet on plasma glucose and enzyme-related indexes of lambs

As shown in Table 6, the content of CK in group B was significantly higher than group CK1 increased by 92.86% (*p* < 0.05); AST in groups A and B was significantly higher than group CK1 increased by 59.03%,56.43%, respectively (*p* < 0.05); ALT groups A and B was significantly higher than group CK1 and other experimental groups increased by 69.74, 73.03%, respectively (*p* < 0.05); there was no significant effect on the Glu and LDH contents among the groups (*p* > 0.05).

**Table 6:** Effects of HBS substituting different proportion of basal diet on plasma glucose and enzyme-related indexes of lambs.

Items	Groups							
	CK1	CK2	A	B	C	D	SEM	*P*
Glu mmol/L	5.88	6.16	5.26	6.47	6.38	6.19	0.127	0.061
CK U/L	0.14^b^	0.18^ab^	0.24^ab^	0.27^a^	0.15^b^	0.15^b^	0.084	0.020
LDH U/L	4011.56	3953.76	4269.75	4269.75	3680.15	3868.97	107.299	0.597
AST U/L	10.35^c^	12.37^abc^	16.46^a^	16.19^ab^	10.53^c^	11.22^bc^	0.756	0.032
ALT U/L	3.04^b^	3.71^ab^	5.16^a^	5.26^a^	2.59^b^	2.55^b^	0.456	0.007

### 3.6 Effects of HBS substituting different proportion of basal diet on relevant indexes of plasma lipid metabolism in lambs

As shown in Table 7, the content of TC in groups A and C was significantly lower than groups CK1 and CK2 (*p* < 0.05); HDL-c in group D was extremely significantly higher than groups CK1 and CK2 increased by 36.11, 40.00%, respectively (*p* < 0.01); and there was no significant effect on the LDL-c and TG contents among the groups (*p* > 0.05).

**Table 7:** Effects of HBS substituting different proportion of basal diet on relevant indexes of plasma lipid metabolism in lambs.

Items	Groups							
	CK1	CK2	A	B	C	D	SEM	*P*
TC mmol/L	2.38^a^	2.28^a^	1.67^b^	2.05^ab^	1.76^b^	2.14^ab^	0.074	0.023
HDL-c mmol/L	0.72^Bb^	0.70^Bb^	0.69^Bb^	0.87^ABa^	0.87^ABa^	0.98^Aa^	0.059	<0.001
LDL-c mmol/L	0.88	0.85	0.79	0.74	0.54	0.68	0.036	0.059
TG mmol/L	0.74	0.66	0.45	0.66	0.59	0.55	0.028	0.054

### 3.7 Effects of HBS substituting different proportion of basal diet on plasma immune-related indexes of lambs

As shown in Table 8, the content of IFN-γ in group C was significantly lower than groups CK1 and CK2 decrease by 20.09, 24.95%, respectively (*p* < 0.05); there was no significant effect on the content of IL-1β, IL-2β, and CD4 among the groups (*p* > 0.05).

**Table 8:** Effects of HBS substituting different proportion of basal diet on plasma immune-related indexes of lambs.

Items	Groups							
	CK1	CK2	A	B	C	D	SEM	*P*
βIL-1β ng/L	29.05	28.59	24.35	27.06	23.35	27.24	0.648	0.050
βIL-2β ng/L	34.77	37.40	35.34	37.33	31.12	34.82	0.852	0.309
IFN-γ ng/mL	86.24^a^	82.89^ab^	74.77^bc^	82.83^ab^	69.02^c^	79.23^abc^	1.621	0.015
CD4 ng/mL	259.43	250.50	217.10	244.04	190.09	227.64	7.330	0.060

### 3.8 Effects of HBS substituting different proportion of basal diet on plasma nitrogen metabolism of lambs

As shown in Table 9, the content of TP in groups A and C was significantly lower than group CK1 (*p* < 0.05); there was no significant effect on BUN content between the groups (*p* > 0.05).

**Table 9:** Effects of HBS substituting different proportion of basal diet on plasma nitrogen metabolism of lambs.

Items	Groups							
	CK1	CK2	A	B	C	D	SEM	*P*
TP g/L	66.69^a^	62.48^abc^	58.54^bc^	61.59^abc^	56.32^c^	63.27^ab^	0.948	0.018
BUN mmol/L	6.86	8.11	6.99	7.55	7.75	8.52	0.184	0.060

### 3.9 Effects of HBS substituting different proportion of basal diet on growth performance of lambs

As shown in Table 10, average daily weight gain, body length, chest circumference, chest depth, chest width, ear length, tube circumference, cross section in each group did not have a group C height was significantly higher than group CK2 (*p* < 0.05); group B ear width was significantly higher than groups CK1, C and D (*p* < 0.05).

**Table 10:** Effects of HBS substituting different proportion of basal diet on growth performance of lambs.

Items	Groups							
	CK1	CK2	A	B	C	D	SEM	*P*
Average daily weight gain g/d	92.00	134.22	112.44	104.89	114.89	101.89	8.97	0.84
Dead weight loss %	20.00	20.00	6.67	13.33	6.67	13.33		
Height cm	4.93^ab^	2.2^b^	5.33^ab^	4.47^ab^	5.93^a^	5.2^ab^	0.47	0.27
Body length cm	3.07	3.73	4.73	4.13	4.93	4.20	0.42	0.84
Chest circumference cm	11.00	14.40	10.53	9.27	14.93	11.20	0.83	0.29
Chest depth cm	2.73	2.20	2.87	2.40	3.20	3.07	0.28	0.90
Chest width cm	0.47	0.40	1.33	1.00	0.73	1.26	0.22	0.75
Perimeter cm	0.40	0.33	0.40	0.53	0.47	0.27	0.07	0.89
Ear length cm	1.93	1.60	1.80	2.27	1.67	1.53	0.15	0.76
Ear width cm	0.33^b^	0.8^ab^	0.8^ab^	1.2^a^	0.4^b^	0.4^b^	0.09	0.04
Crucifixion cm	4.93	4.20	5.07	4.80	5.13	3.67	0.46	0.94

## 4 Discussion

Milk components are divided into two parts, water and solids, solids include a variety of substances such as fat, protein, lactose, minerals and vitamins, and the amount of various components of solids affects the milk quality (14). Lactose, milk fat percentage, total protein, albumin etc. as regular nutrients in sheep milk play a vital role in the growth and development of lambs. Saidi and Omar (15) and Badran et al. (16) replaced regular wheat hay with hydroponically grown barley seedling in the diets of lactating ewes and found that there was no effect on the feed intake, body weight, milk yield and milk composition of the ewes. Samir et al. (12) replace the basal diet with 25% hydroponic feed in the diet of Holstein dairy cows, which can improve the dry matter intake, daily gain and body condition score of dairy cows, as well as milk yield, milk fat percentage and TS content in milk, which is consistent with the results of this study. In this experiment, addition of different proportions of HBS significantly increased lactose, milk fat percentage, TS, TP and ALB contents in sheep milk, which may be related to the fact that the present experiment was conducted by substituting hydroponically grown barley seedlings for different proportions of the basal diet, under the same dry matter conditions, hydroponic feed contains a large amount of protein and amino acid gas, which can improve the digestion and absorption of ruminants, and contains more bioactive substances such as vitamins and polyphenols (17), thus having a positive impact on milk composition. Studies have shown that wheat seedling flour contains a large number of flavonoids and related compounds with strong antioxidant activity (18). In this experiment, the addition of different ratios of hydroponically grown barley seedlings significantly provided GSH-px content in sheep milk, which may be attributed to the antioxidant effect of hydroponically grown barley seedlings containing polyphenolic actives that can scavenge free radicals in the organism and prevent them from damaging the organism (19).

Most of the nutrients required by lambs in the pre-development period come from breast milk, which has the advantages of high elimination and comprehensive nutrition, and is an important source of nutrients during the period from birth to weaning. Blood biochemical level reflects the nutrition and organ metabolism of protein, amino acid, sugar and lipid in the animal body, and blood biochemical indexes are affected by the health condition of the body on one hand, and the nutritional status of the body as well as the nutritional level of the diet on the other hand. In this experiment, with the increase of hydroponic barley seedling substitution, the contents of CK, AST, and ALT had a tendency to increase in all groups, among which, the contents of AST and ALT in group A were significantly higher than those in group CK1; and the contents of CK in group B were significantly higher than group CK1, which means that the substitution of different ratios of basal diets by hydroponic barley seedling had a positive influence on the metabolism of the liver. Plasma cholesterol content can reflect the lipid metabolism of the body (20) and cholesterol can be divided into LDL cholesterol and HDL cholesterol (21). Studies have shown that hydroponically grown barley seedlings are a good source of vitamins, with each 1 kg of hydroponically grown barley seedlings containing 62.4 mg of vitamin A and 1.05 mg of free folic acid (22, 23). Raeisi et al. (24) used 7, 14, and 21% of the body lipids in the diets of sheep with hydroponically grown barley seedlings (fresh weight) in equal proportions to replace some of the barley kernels, and the differences in BUN content between the groups were not significant as the amount of hydroponically grown barley seedlings replaced increased. Studies in diabetic patients and mouse animal models have found that the nutrients in barley seedling can reduce blood lipids, alleviate liver function damage and improve oxidative stress levels. Jiaqiang et al. (25). found that supplementation of hydroponic barley seedlings with restricted basal diet can significantly reduce the blood total cholesterol content and abdominal fat percentage of seeded geese, and hydroponic barley seedlings may regulate the body’s fat deposition capacity by reducing blood lipid, which is consistent with the results of blood biochemical indexes in this study. In this experiment, with the increase of hydroponic barley seedling substitution, HDL-c content significantly increased, TP and TC content decreased, and the difference of BUN content was not significant among groups, which may be related to the vitamins contained in hydroponic barley seedling, and folic acid has an important role in regulating the metabolism of lipids and other metabolisms, whereas the content of folic acid in breast milk may be insufficient to promote the protein synthesis and the metabolism of sugar and lipids in lambs (26, 27), and this may be the reason for the decrease of TP and TC content. The reason for the decrease in TP and TC content. A complex network of antioxidant enzymes and non-enzymatic antioxidants that effectively scavenge reactive oxygen species exist in mammalian cells (28). In the present experiment, hydroponically grown barley seedlings in place of different proportions of basal diets increased plasma GSH-px levels and decreased serum MDA levels in lambs. The results of the experiment may be attributed to the fact that the polyphenol content contained in hydroponically grown barley seedlings indirectly increased the content of relevant antioxidant enzymes in the ewe’s organism, thus improving the antioxidant capacity of the organism (29).

Growth and development of young lactating animals are influenced by various factors, including breed (genetic factors), maternal effects (newborn weight), and feeding management (breeding environment, diet composition, and dietary nutrition level). The addition of 23% hydroponic barley seedlings to the diet of laying hens breeders can increase the growth rate of breeders, promote the growth and egg production rate of laying hens, and enhance the overall economic efficiency of laying hens (30). The addition of 5% barley malt meal to the diet of lactating piglets increased feed utilization by 7.1% (31).In this experiment, the optimal daily weight gain of lambs in group CK2 may be due to the reduction in the number of lambs, which resulted in an increase in the feed intake of individual lambs and an increase in the weight of lambs in comparison to the replacement group. However, in terms of overall substitution level, group C had the best average daily weight gain, which may be due to the low dry matter and starch content of hydroponically grown barley seedling, in which starch can be mostly decomposed and metabolized to soluble polysaccharides, and the addition of 15% hydroponically grown barley seedling can be used to meet the metabolic and energy needs of the lambs’ organisms. From the analysis of the mortality rate, the mortality rate of each experimental group was lower than that of the control group, and the mortality rate of groups A and C was the lowest. The mortality rate was significantly lower in all the test groups than in the control group, and the lowest mortality rate was achieved with the use of 5 and 15% hydroponically grown barley seedlings in place of the basal ration. Since the mortality rates were within the permissible normal range, there was no mortality due to external factors.

## 5 Conclusion

Under the conditions of this test, HBS instead of 5–15% of the basal diet were able to improve milk quality and alleviate oxidative stress in the body of lactating Hu ewes under the conditions of this experiment.

## Data availability statement

The original contributions presented in the study are included in the article/supplementary material, further inquiries can be directed to the corresponding author.

## Ethics statement

The animal study was approved by the Feed Research Institute, Xinjiang Academy of Animal Sciences. The study was conducted in accordance with the local legislation and institutional requirements.

## Author contributions

YM: Writing – original draft, Writing – review & editing. TG: Conceptualization, Data curation, Investigation, Writing – original draft. ZZ: Methodology, Software, Writing – review & editing. GA: Formal analysis, Project administration, Writing – review & editing. YJ: Formal analysis, Project administration, Writing – review & editing. YT: Funding acquisition, Resources, Writing – review & editing. LH: Methodology, Project administration, Writing – review & editing.
